# Non-chemotherapy adjuvant agents in TP53 mutant Ewing sarcoma

**DOI:** 10.1038/s41598-023-40751-z

**Published:** 2023-09-01

**Authors:** Jin-Ah Kim, Kenneth A. Crawford, Piero A. Spada, Leah R. Martin, Jiaqi Zhang, Rain Wong, Joel M. Reid, Clinton F. Stewart, Timothy M. Frank, Qianqian Liu, Joel E. Michalek, Charles Keller

**Affiliations:** 1 Children’s Cancer Therapy Development Institute, 9025 NE Von Neumann Drive Ste 110, Hillsboro, OR 97006 USA; 2 Department of Molecular Pharmacology and Experimental Therapeutics, Mayo Clinic, Rochester, MN 55905 USA; 3 Department of Pharmacy and Pharmaceutical Sciences, St. Jude Children’s Research Hospital, Memphis, TN 38105-2794 USA; 4 Department of Epidemiology and Biostatistics, University of Texas Health Science Center, San Antonio, TX 78229 USA

**Keywords:** Cancer, Cancer therapy, Chemotherapy

## Abstract

Ewing sarcoma (EWS) is a malignant tumor arising in bone or soft tissue that occurs in adolescent and young adult patients as well as adults later in life. Although non-metastatic EWS is typically responsive to treatment when newly diagnosed, relapsed cases have an unmet need for which no standard treatment approach exists. Recent phase III clinical trials for EWS comparing 7 vs 5 chemotherapy drugs have failed to improve survival. To extend the durability of remission for EWS, we investigated 3 non-chemotherapy adjuvant therapy drug candidates to be combined with chemotherapy. The efficacy of these adjuvant drugs was investigated via anchorage-dependent growth assays, anchorage-independent soft-agar colony formation assays and EWS xenograft mouse models. Enoxacin and entinostat were the most effective adjuvant drug in both long-term in vitro and in vivo adjuvant studies. In the context that enoxacin is an FDA-approved antibiotic, and that entinostat is an investigational agent not yet FDA-approved, we propose enoxacin as an adjuvant drug for further preclinical and clinical investigation in EWS patients.

## Introduction

Ewing sarcoma (EWS) is a highly malignant tumor of bone and soft tissue that occurs in a bimodal distribution in children, adolescents, and young adults but also older adults^1^. Despite the use of combination chemotherapy resulting in a marked improvement in patient outcomes, metastasis or primary site recurrence usually occur within the first few years after treatment^2^. The relapse of EWS can happen as late as three decades after therapy in both sexes^3,4^. Cases of late recurrence have been reported after pregnancy in women^3,4^.

Although multi-agent chemotherapy and multidisciplinary care has considerably elevated the survival rate of patients with localized EWS to nearly 70%, survival in a recurrent or metastatic disease is unacceptably low at < 20%^5^. Furthermore, addition of topotecan, additional cyclophosphamide and additional vincristine to interval compressed chemotherapy with vincristine, doxorubicin, cyclophosphamide, ifosfamide and etoposide had no benefit to survival for patients with untreated, non-metastatic EWS^6^. Given the non-trivial 5–6% risk of secondary malignancy (i.e. myelogenous leukemia) already attributable to standard of care chemotherapy for EWS^7^, exploring non-chemotherapy adjuvants is warranted.

The most aggressive and often most refractory subsets of Ewing sarcoma have *TP53* mutations in approximately 7% of cases (range 3–14%, as discussed in^8^). The *TP53* mutant subset has a propensity for radiation resistance^8^. *STAG2* mutations occur in approximately 17% of cases^9^ and portend a similarly poor prognosis^10^. Treatment options for these aggressive subsets of patients are a particularly unmet clinical need.

**Figure 1:**
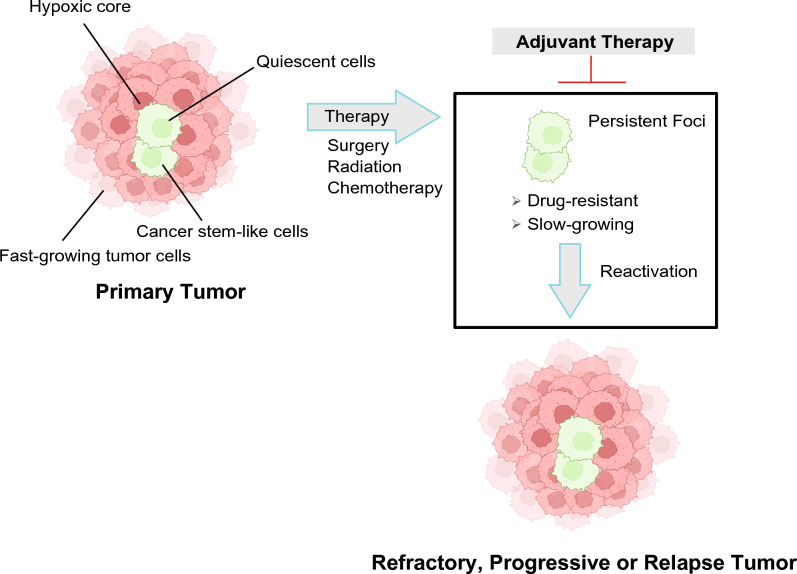
Working model and rationale for an adjuvant therapy approach to prevent progression of Ewing sarcoma.

Goldie and Coldman presented one theory for chemotherapy effectiveness, resistance and relapse in that increased cytoreduction will reduce the number of mutation-driven resistant, re-emergent tumor cell clones^11^. Norton and Simon proposed a complementary hypothesis that chemotherapy effectiveness is related to tumor cell growth rate^12^. A subsequent related hypothesis describes slow-dividing, tumor repopulating cells: these cancer cell subpopulations (also known as, cancer stem cells) are inherently chemotherapy-resistant due to their stem cell-like drug efflux pumps and ability to be quiescent in periods of chemotherapy-related stress (Fig. 1)^13^. Each of these hypotheses imply the same practical goal: improved log cell kill at each chemotherapy cycle is valuable to patient long term survival, especially if slow-dividing cells can be effectively targeted.

Here we investigated 3 preclinically- and clinically-investigated non-chemotherapy adjuvant drug candidates for their potential to improve chemotherapy effectiveness, using etoposide as a topoisomerase II inhibitor causing double-stranded DNA breaks^14,15^ as our chemotherapy model drug. TK216 (also known as, ONCT-216) is an analog of YK-4-279 that has recently been studied in EWS with vincristine in a phase 2 clinical trial together (NCT05046314)^16^, but has been since deprioritized by Oncternal Therapeutics and is now also thought to be a microtubule inhibitor^17^. Mechanistically, YK-4-279 (and presumably TK-216) binds directly with the pathognomonic fusion-mediate chimeric transcription factor and tumor maintenance driver EWS-FLI1^18–21^ to inhibit its interaction with RNA helicase A (RHA)^22^. YK-4-279 has been shown to induce apoptosis and growth inhibition of EWS tumors^22,23^. Entinostat is a histone deacetylase inhibitor that reveals high potency against Type I HDACs^24^ and has preclinical activity against EWS^17^. A Phase 1 clinical study for entinostat was performed in patients with advanced solid tumors or lymphoma (NCT00020579), and single agent activity was demonstrated in an EWS patient^25^. Enoxacin is an oral fluoroquinolone antibacterial agent that was shown to enhance TRBP/DICER dependent miRNA maturation^26^, leading to tumor regression^27,28^ and EWS tumor stem cell depletion^29,30^. The miRNA maturation led by enoxacin inhibited cancer stem cell self-renewal and tumor maintenance^30^.

These 3 adjuvant drugs were tested with the chemotherapy etoposide in short-term and long-term two-dimensional (2D) cell inhibition assays, soft-agar colony formation assays and in vivo EWS mouse models. In these studies, enoxacin and entinostat were found to be the most effective drug candidates in both long-term in vitro and in vivo adjuvant studies. We propose enoxacin as a potential adjuvant drug for further preclinical and clinical EWS studies based on the basis of its FDA approval status.

## Results

### Long-term effects of adjuvant drugs in 2D cell culture

**Figure 2:**
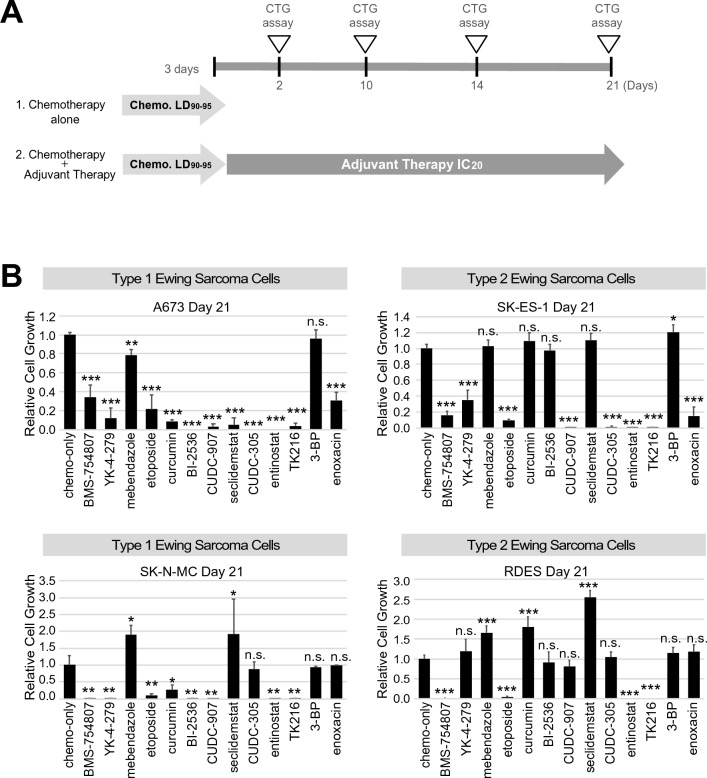
Anchorage-dependent adjuvant therapy assay in Ewing sarcoma cells. (**A**) Schematic design of assay. (**B**) Adjuvant assay results in cell lines A673 and SK-N-MC (type 1 EWS) as well as SK-ES-1 and RDES (type 2 EWS). The cells were treated with adjuvant drug candidates for 21 days after 3-day etoposide treatment. The surviving cells were measured using CTG. Statistical analysis was performed using two sided *t* test. ***p < 0.001; **p < 0.01; *p <0.05 versus chemo-only. Chemo, chemotherapy (etoposide). All experiments included at least three replicates.

**Figure 3:**
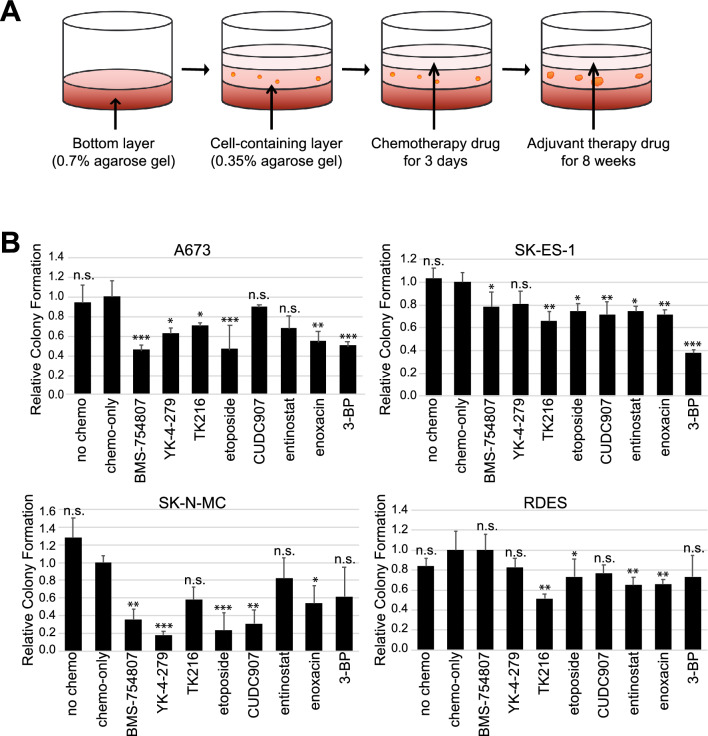
Anchorage-independent soft agar colony formation assay in Ewing sarcoma cells. Soft agar assay was performed in A673, SK-N-MC, SK-ES-1, and RDES EWS cell lines. Cells were treated with 1.6 µM of etoposide for 3 days, then followed by adjuvant drug candidate treatment. (**A**) Diagram of 8 week soft agar colony formation assay. (**B**) Cell colonies were counted from soft agar assay in four Ewing sarcoma cell lines after adjuvant drug treatments for 8 weeks. Statistical analysis was performed using two sided *t* test. ***p < 0.001; **p < 0.01; *p <0.05 versus no chemo. Chemo, chemotherapy. 3-BP, 3-bromopyruvate. All experiments included at least three replicates.

To investigate the long-term effect of the adjuvant drug candidates, an anchorage-dependent 2D cell culture cell growth/viability assay was performed over 24 days. The design of the assay was to “debulk” tumor cell mass over 72 hr with a EWS-specific chemotherapy (etoposide) followed by application of the adjuvant drugs. EWS cell lines A673, SK-N-MC, SK-ES-1 and RDES (Table 1) were treated with etoposide using 1.6 µM at a lethal dose (LD) 90–95% for 3 days. Thereafter, candidate adjuvant drugs were added at concentrations of either the IC_20_ (990 nM for entinostat) or a clinically relevant concentration (C_steady-state_ 3.9 µM for TK-216 (J. Toretsky, p.c.), C_max_ 9.2 µM for enoxacin^31,32^) for 21 days (Fig. 3A,B). As a control, lower dose etoposide (427 nM) was used at the IC20 as an alternative to the non-chemotherapy adjuvant agents. The long-term adjuvant assay showed that TK216, etoposide, and entinostat were statistically active adjuvant drugs across all four EWS cell lines (Fig. 2).

**Table 1:** EWS cell lines, EWSR1 fusion type and TP53 status.

Cell line	Fusion type	Other features (TP53, CDKN2A and STAG2 status)	Reference (PMID)
A673, SK-N-MC, TC71	Type 1 (*EWSR1* ex7–*FLI1* ex6)	A673 (*TP53* p.A119fs; *CDKN2A* del(1a,1b,2,3); *STAG2* WT)	26776507, 8223458, 21926473, 8040301, 12432241, 19212622, 24129240, 18757425, 25223734
		SK-N-MC (*TP53* p.M1_T125Del; *CDKN2A* WT; *STAG2 p.M1_R546Del*)	
		TC71 (*TP53* p.R213*; *CDKN2A* del(1b,2,3); *STAG2* WT)	
SKES-1, RDES	Type 2 (*EWSR1* ex7–*FLI1* ex5)	SKES-1 (*TP53* C176F; *CDKN2A* WT; *STAG2* p.Q735*)	26776507, 8223458, 8040301, 12432241, 24129240, 18757425, 25223734
		RDES (*TP53* p.R273C; *CDKN2A* WT; *STAG2* WT)

### Effect of adjuvant drugs in soft agar colony formation assay

Anchorage-independent growth is the feature of the transformed cells, a hallmark of carcinogenesis^33^ and a characteristic of tumor repopulating cells. To further evaluate the effect of the adjuvant drug candidates in EWS, we performed soft-agar colony formation assays using EWS cell lines A673, SK-N-MC, SK-ES-1 and RDES. The adjuvant drugs were dosed for 8 weeks at concentrations of either IC_20_ or C_max_ following 1.6 µM etoposide treatment for 3 days (Fig. 3A). Enoxacin and low-dose etoposide at the IC20 were identified as the most effective adjuvant drug across all four EWS cell lines. The reduction of colony formation in enoxacin and etoposide treatments was ranged from 28–46% and 26–77% in four cell lines with statistically significance (*p* ≤ 0.05) (Fig. 3B). Soft-agar colony formation was also significantly suppressed in at least two EWS cell lines by TK216 and entinostat (Fig. 3B).

### Effect of adjuvant drugs for in vivo mouse study

**Figure 4:**
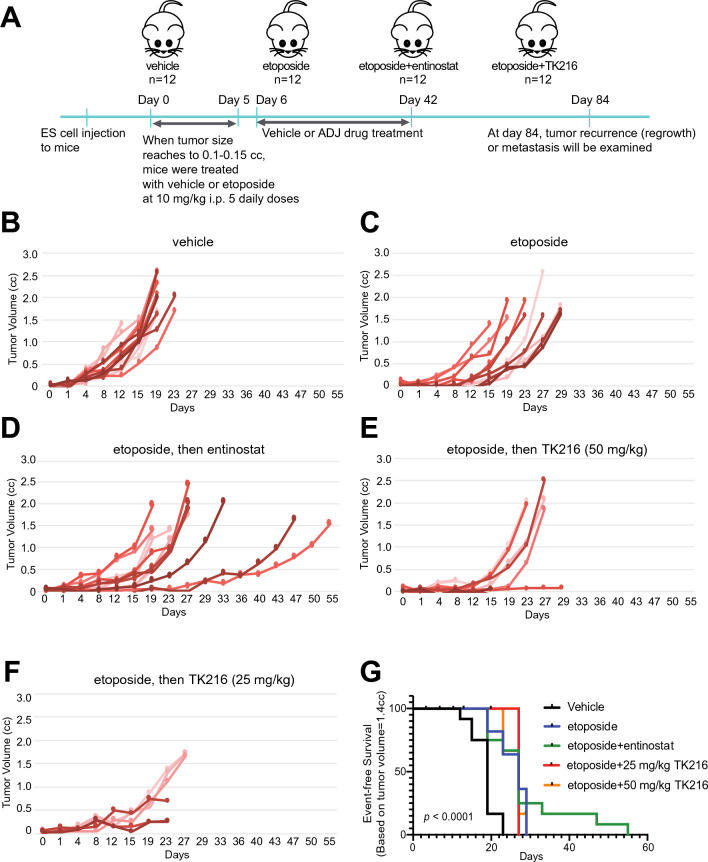
Effect of entinostat and TK216 for adjuvant therapy in vivo mouse study. SK-N-MC EWS cells harboring *TP53* and *STAG2* mutations were injected in the right leg of 6-week old male (n = 4) and female (n = 8) NOD/SCID/IL2gr-null mice in each treatment group. When tumor size reached to 0.1–0.5 cc, mice were treated with 10 mg/kg etoposide for 5 days followed by the adjuvant drugs for 37 days dosed at the estimated human drug exposures described in “Materials and methods”. (**A**) Diagram of mice study. Individual tumor volumes in (**B**) vehicle, (**C**) etoposide-only, (**D**) entinostat following etoposide, (**E**) TK216 (50 mg/kg) after etoposide treatment, and (**F**) TK216 (25mg/kg) following etoposide. (**G**) Kaplan-Meier plot represents event-free survival. The event was defined as when tumor reached to 1.4 cc. Overall *p*-values were calculated by the log-rank test. *P *< 0.001 for each treatment group’s comparison to vehicle only.

**Figure 5:**
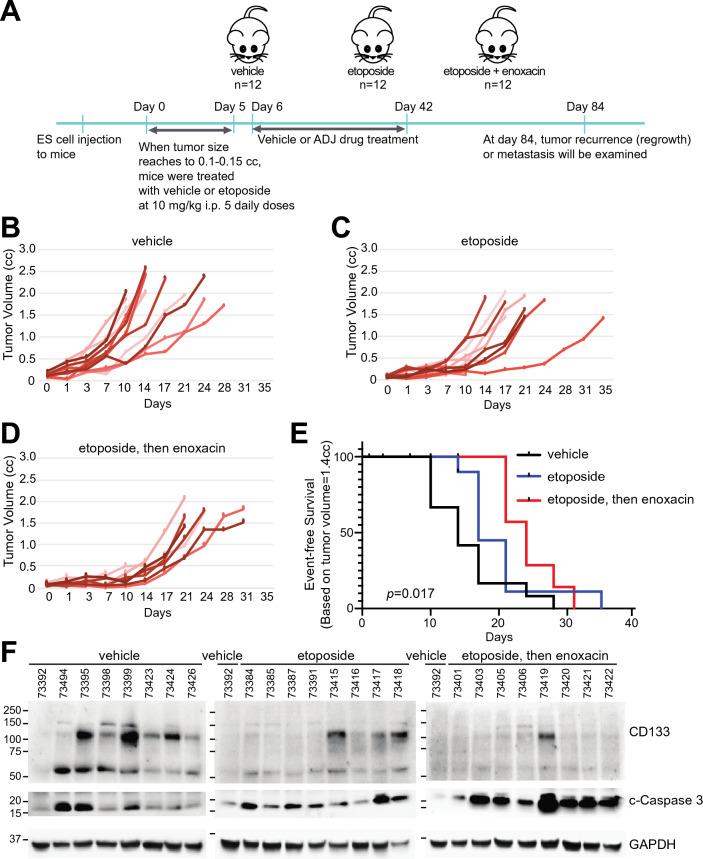
Effect of enoxacin for adjuvant therapy in vivo mouse study. Mouse study was performed as described in Fig. 5. (**A**) Diagram of mice study. Individual tumor volumes in (**B**) vehicle, (**C**) etoposide-only and (**D**) enoxacin following etoposide treatment. (**E**) Kaplan-Meier plot represents event-free survival. The event was counted when tumor reached 1.4 cc. Overall *p*-values were calculated using the log-rank test. *P *= 0.014 for etoposide then enoxacin group compared to vehicle only. **(F)** The expression of CD133 and cleaved (c)-caspase 3 in EWS xenograft tumors from vehicle, etoposide-only, or enoxacin after etoposide treated groups. Eight protein samples in each group were used for western blot. To compare the protein intensity among immunoblots, one vehicle sample was applied in the first lane of each blot. The relative expression of CD133 and c-caspase 3 was shown in the graph of densitometry analysis.

Next we tested entinostat, TK216, and enoxacin for their adjuvant efficacy using in vivo mouse models (Figs. 4 and 5). Drug doses were selected to be most similar between humans and mice (Tables 2 and 3). For engraftment, SK-N-MC EWS cells were injected in the right leg of 6 week-old male (n = 4) and female (n = 8) NOD/SCID/IL2gr-null mice in each treatment group. When the tumor size reached 0.1–0.15 cc, mice were treated with 10 mg/kg etoposide for 5 days followed by adjuvant drugs for 37 days dosed at the estimated human drug exposures described in “Materials and methods”. Tumor growth rates were then measured. Two mice studies were performed to evaluate the adjuvant drugs. First, entinostat and TK216 were used as adjuvant drugs (Fig. 4A–G). Next, enoxacin was tested as the adjuvant drug (Fig. 5A–F). Entinostat treatment following etoposide was effective for tumor regression compared with the vehicle group (*p* < 0.001), but not when compared with the etoposide-only treated group (*p *= 0.57) (Fig. 4B,C,G). TK216 caused toxicity >10 % (weight loss) for both doses of 25 mg/kg QD and 50 mg/kg QD with 50 % treatment-related mortality. The efficacy of TK216 was similar to but less sustained than entinostat (Fig. 4G). Overall *p*-value was < 0.001 in the mouse study (Fig. 5E–G). Enoxacin inhibited the tumor progression most efficiently compared with both the vehicle group (*p* < 0.05) and etoposide-only treated group (*p *= 0.4) showing the overall *p*-value as 0.017 (Fig. 5B–E). For tumors harvested either at day 84 or when tumor volume reached 1.4 cc, enoxacin treatment following etoposide inhibited expression of a cancer stem cell marker CD133 in EWS^34^, and increased cleaved-caspase 3 which indicates the elevated apoptosis compared with that of the vehicle or etoposide-only groups (Fig. 4). All regimens were well-tolerated by body weight monitoring (Supplementary Figs. 1 and 2).

**Table 2:** Etoposide dosing consideration from the literature.

	Human				Mouse			
Agent	Dose	AUC^^^	Fu,p	AUCu	Dose	AUC^#^	Fu,p	AUCu
Etoposide	100 mg/m^2^ I.V.	84,000 hr-ng/ml	0.049^36^	4120 hr-ng/ml	10 mg/kg I.P.	14,300 hr-ng/ml^37^	0.423^36^	6050 hr-ng/ml

Fu,p fraction unbound in the plasma. *AUCu* area under the concentration-time curve for unbound drug.

^^^Cmax 16.3 ± 3.7 µg/ml (27.7 µM)^38^.

^#^Cmax 32.1 ± 1.9 µg/mL (54.54 µM) for the IV dose, with IP=IV assumed.

**Table 3:** Etoposide and adjuvant doses, route, interval and literature references.

Drug	Target	Dose	Administration method
Entinostat	HDAC 1, 2, 3	5.5 mg/kg, QD	Oral gavage^39,40^
Enoxacin	TARBP2	200 mg/kg, QD	Oral gavage^41^
TK216	EWSR1-FLI1	25 or 50 mg/kg, QD	I.P.^42^

## Discussion

Although newly diagnosed EWS patients may expect long-term benefit from upfront chemotherapy, metastatic and relapsed EWS patients have consistently poor outcomes. Here we evaluated 3 drug candidates via long-term in vitro and in vivo assays for effectiveness as adjuvants to chemotherapy in EWS. While in vitro assays were useful first evaluations, the most informative results came from EWS xenograft mouse model studies.

In some animals and overall, entinostat suppressed tumor growth in a subset of animals and extended overall xenograft animal survival after etoposide compared to etoposide alone. Enoxacin had a more consistent ability to suppress tumor growth and extend overall xenograft animal survival after etoposide compared to etoposide alone – albeit with a similar effect on time to event compared to entinostat. As described in the Introduction, the mechanism underlying enoxacin activity as a chemotherapy adjuvant and tumor repopulating cell depletion has been well-described previously^29,30^. Given that enoxacin is an FDA-approved antibiotic that might be repurposed, and entinostat is an agent not yet FDA approved, we have set future sights on this agent for further preclinical investigation.

Like many pilot studies, our investigation has potential limitations and notable future directions. For example, increasing cohort size may improve power and statistical significance. Studying additional patient-derived xenograft models (e.g. more independent biological replicates) would also extend the overall value of these preclinical studies in clinical trial concept development. The expression of CD133 and cleaved-caspase 3 in mouse tumors treated with etoposide and enoxacin suggests that the sequential treatment of the drugs suppress stem cell formation inducing cell death. Further investigation in the expression of CD133 and cleaved-caspase 3 with other treatments such as etoposide with entinostat will also be of interest in future studies. Assessment of CD133+ tumor cells by FACS at a fixed timepoint or terminal endpoint in animal studies would also add mechanistic insights.

In conclusion, enoxacin was identified as an efficient adjuvant drug by long-term 2D cell culture, soft agar colony formation and in vivo studies. Taken altogether with its FDA approval status, we propose enoxacin or a fluoroquinolone with fewer drug interacts^35^ as an adjuvant agent for additional preclinical animal studies then possible clinical trial investigation in EWS.

## Materials and methods

### Cell lines

A673 (cat# CRL-1598), SK-N-MC (cat# HTB-10), SK-ES-1 (cat# HTB-86), and RD-ES (cat# HTB-166) EWS cells were purchased from American Type Culture Collection (ATCC, Manassas, VA). A673 was cultured in DMEM medium with 10% fetal bovine serum (FBS) and 1% Penicillin-Streptomycin (PS). SK-N-MC was maintained in EMEM medium with 10% FBS and 1% PS. SK-ES-1 was grown in McCoy’s 5A medium with 10% FBS and 1% PS. RD-ES was cultured in RPMI medium with 10% FBS and 1% PS. TC71 (cat# GM11654) was purchased from Coriell Institute (Camden, NJ) and cultured in EMEM medium 10% FBS and 1% PS.

### 2D cell culture adjuvant assay

Twenty thousand EWS cells were seeded in 48-well plates. The next day, EWS cells were treated with 1.6 µM etoposide (LD_90-95_) for 3 to 5 days, then candidate adjuvant therapy drugs were added at concentrations of either IC_20_ or Cmax for 21 days (Table 1). The adjuvant drug candidates tested were etoposide (Selleckchem, Houston, TX, cat# S1225), entinostat (Selleckchem, cat# S1053), enoxacin (MedChemExpress, Monmouth Junction, NJ, cat# HY-B0268A) and TK216 (Selleckchem, cat# S9718). Cell growth was examined by the CellTiter-Glo proliferation assay (Promega, Madison, WI, cat# G9243).

### Soft agar colony formation assay

Five thousand EWS cells were suspended in growth medium containing 0.35 % SeaPlaque agarose and then plated on 0.7 % base agar in 6-well plate. After 24 hours, EWS cells were treated with 1.6 µM etoposide (LD_90-95_) for 3 days, then adjuvant drug candidates were added at concentrations of either the IC_20_ or clinical Cmax for 8 weeks (see “Results”). EWS cells were treated with TK216, entinostat, enoxacin or etoposide. Drugs were added every 3.5 days (twice a week). The colonies were quantified from examination of n = 8 random fields of view in each well using the confocal microscope (Zeiss, Oberkochen, Germany, #LSM-800) at the endpoint of the experiment.

### Western blotting

Frozen tumor tissues were homogenized in the presence of liquid nitrogen using a mortar and pestle kit (Cole-Parmer, Vernon Hills, IL, cat# UX-63100-61). Protein extraction was performed by adding RIPA buffer supplemented with Halt protease and phosphatase inhibitor cocktail (ThermoFisher, cat# 78440). Protein samples were prepared in 4X SDS loading buffer (Thermofisher, cat# NP0007) and 1X reducing reagent (ThermoFisher, cat# NP0004). After running the SDS-PAGE gel, proteins were transferred on PVDF membrane with 0.2 µm pore size (Bio-Rad, Hercules, CA, cat# 162-0177) by wet transfer method at 90 V for 90 mins. The following antibodies were used at the indicated dilutions: CD133 (Cell Signaling Technology (CST), Danvers, MA, cat# 64326, 1:1,000), Cleaved Caspase-3 (CST, cat# 9661, 1:500) and GAPDH (CST, cat# 2118, 1:1000). Primary antibodies were incubated at 4 °C for overnight. Secondary antibodies were incubated at room temperature for one hour. Images were taken using the IVIS Lumina imager (Perkin-Elmer, Waltham, MA) followed by ECL reaction for 5 min.

### Mouse studies

Host animals for xenografts were 6 week-old male and female NOD/SCID/IL2gr-null mice purchased from The Jackson Laboratory (Bar Harbor, MA, stock # 005557). Mice were inoculated with 5x10^5^ of SK-N-MC cells to the gastrocnemius after gastrocnemius injury was induced by 50 µl of 2.5 µM cardiotoxin for 24 hr. SK-N-MC was chosen as a representative EWS cell culture as it harbors the typical Type I fusion of *EWSR1* to *FLI1* as well as a *TP53* mutation. When tumor volume reached 0.1–0.15 cc, 10 mg/kg of etoposide (Selleckchem, cat# S1225; Table 2) or vehicle was administered for 5 days via daily i.p. injection. After 1 day rest, mice were treated with the adjuvant drug candidates entinostat (Selleckchem, cat# S1053, 5.5 mg/kg, QD, oral gavage), enoxacin (MedChemExpress, cat# HY-B0268A, 100 mg/kg, QD, oral gavage; Table 3) and TK216 (Selleckchem, cat# S9718, 25 mg/kg or 50 mg/kg, QD, i.p injection; Table 3). The drugs were dissolved in 5% DMSO, 40% PEG300, 5% Tween 80 and 50% of ddH_2_O that were used as a vehicle in all mice studies. The adjuvant drugs were dosed until day 42, and primary tumor size and weight of the mouse were measured twice a week with digital calipers during treatment. Tumor progression was defined as a 25% increase in volume. Animals were grossly examined for post-treatment recurrence or metastasis at the end of the experiment on day 84, or when tumor volume reached the humane endpoint of 1.4 cc. Table 3 summarizes etoposide and adjuvant drug doses used and references for dose selection.

### Animal research

Children’s Cancer Therapy Development Institute (cc-TDI) Institutional Animal Care and Use Committee (IACUC) approved the investigated animal study. The IACUC Federal wide Assurance number is 14-5406A. In animal studies, anesthesia was performed using inhaled isoflurane by means of an anesthesia machine with a scavenger system. For euthanasia, CO_2_ was delivered from a compressed gas cylinder to a chamber that had not been pre-filled. Animals were asphyxiated by CO_2_ inhalation, then cervical dislocation was subsequently performed. All methods were carried out in accordance with relevant guidelines and regulations. All methods are reported in accordance with ARRIVE guidelines (https://arriveguidelines.org).

### Supplementary Information

Supplementary Figures.

## Data Availability

No sequencing data was conducted, and all other data are given in this manuscript.
