# Supplementary material for: Non-chemotherapy adjuvant agents in TP53 mutant Ewing sarcoma
Source: Sci Rep. 2023 Sep 1;13:14360. doi: 10.1038/s41598-023-40751-z (PMC10474113; doi:10.1038/s41598-023-40751-z)
Supplement: Supplementary file 1 — Supplementary Figures. [file 41598_2023_40751_MOESM1_ESM.docx]

**Supplementary Material**

**

**Supplementary Figure 1. The weight changes of individual mouse during the drug treatments in vivo mouse study of Figure 5.** Mouse weights were measured as described in Materials and Methods.

**

**Supplementary Figure 2. The weight changes of individual mouse during the drug treatments in vivo mouse study of Figure 6.** Mouse weights were measured as described in Materials and Meth
